# Clinicopathological Characteristics of Serrated Polyposis Syndrome in Korea: Single Center Experience

**DOI:** 10.1155/2015/842876

**Published:** 2015-05-12

**Authors:** Hyung-Keun Kim, Kyung-Jin Seo, Hyun Ho Choi, Sung Soo Kim, Hiun-Suk Chae, Ok-Ran Shin, Chang Hyuck Ahn, Young-Seok Cho

**Affiliations:** ^1^Department of Internal Medicine, Uijeongbu St. Mary's Hospital, College of Medicine, The Catholic University of Korea, Uijeongbu 480-717, Republic of Korea; ^2^Department of Hospital Pathology, Uijeongbu St. Mary's Hospital, College of Medicine, The Catholic University of Korea, Uijeongbu 480-717, Republic of Korea; ^3^Department of General Surgery, Uijeongbu St. Mary's Hospital, College of Medicine, The Catholic University of Korea, Uijeongbu 480-717, Republic of Korea

## Abstract

*Background/Aim.* Serrated polyposis syndrome (SPS) is a rare condition characterized by multiple serrated polyps throughout the colon and rectum. The aim of this study was to evaluate the clinicopathological characteristics of SPS in Koreans.* Methods.* This retrospective analysis of prospectively collected data was performed using information from the endoscopy, clinical records, and pathology database system of Uijeongbu St. Mary's Hospital. Consecutive patients satisfying the updated 2010 World Health Organization criteria for SPS between June 2011 and May 2014 were enrolled.* Results.* Of the 17,552 patients who underwent colonoscopies during the study period, 11 (0.06%) met the criteria for SPS. The mean age of these patients was 55.6 years. Ten patients (91%) were males. None had a family history of CRC or a first-degree relative with SPS. Seven patients (64%) had synchronous advanced adenoma. One patient had coexistence of SPS with CRC that was diagnosed at the initial colonoscopy. Five patients (45%) had more than 30 serrated polyps. One of the patients underwent surgery and 10 underwent endoscopic resection.* Conclusion.* The prevalence of SPS in this study cohort was comparable to that in Western populations. Considering the high risk of CRC, correct diagnosis and careful follow-up for SPS are necessary.

## 1. Introduction

Colorectal cancer (CRC) is one of the most commonly diagnosed cancers worldwide [1]. Its incidence in Asian countries is increasing rapidly, causing a major public health issue [2]. Indeed, in Korea, CRC is the second most common cancer in males and the third most common cancer in females [3]. It is known that the adenoma-carcinoma sequence is the basis for tumorigenesis and progression of CRC [4, 5]. Emerging evidence shows that one-third of CRC arises through the serrated neoplastic pathway, which is characterized by abnormal promoter CpG island hypermethylation and activation of the* BRAF* oncogene with or without microsatellite instability [6]. The precursor lesion in this pathway is a serrated polyp. Serrated polyps of the colorectum are characterized histologically by a serrated, saw-tooth appearance and are classified by the World Health Organization (WHO) into three general categories: hyperplastic polyp (HP), sessile serrated adenomas/polyps (SSA/Ps) with or without cytological dysplasia, and traditional serrated adenomas (TSAs) [7].

Serrated polyposis syndrome (SPS), previously called “hyperplastic polyposis syndrome,” is a rare condition, characterized by multiple serrated colorectal polyps [7]. A recent study from UK showed that SPS appeared to be relatively common (1 : 151) in patients undergoing screening colonoscopy based on fecal occult blood testing [8]. Although the exact risk of CRC in SPS is unknown, the risk of personal and familial CRC is increased in SPS. Published case series and cohort studies have reported rates of CRC from 25 to 50% at first clinical presentation in patients with SPS [9–11]. A retrospective cohort study showed that the cumulative risk of CRC under endoscopic surveillance was 7% in 5 years [9]. Thus, close endoscopic surveillance and adequate management are required to prevent malignant progression.

There are few studies regarding the prevalence or phenotypic characteristics of SPS in Asian countries. The aim of this study was to study the prevalence and clinicopathological characteristics of SPS in Korea.

## 2. Patients and Methods

### 2.1. Patients

We performed a retrospective study in 11 patients with SPS identified at Uijeongbu St. Mary's Hospital between June 2011 and May 2014. The following WHO criteria were used to identify SPS: (1) at least five serrated polyps proximal to the sigmoid colon with two or more of them being >10 mm, (2) any number of serrated polyps proximal to the sigmoid colon in an individual who has a first-degree relative with serrated polyposis, or (3) >20 serrated polyps of any size, but distributed throughout the colon [7]. We excluded cases with a family history of familial adenomatous polyposis (FAP), hereditary nonpolyposis colorectal cancer (HNPCC), or familial colorectal cancer, surgical resection of the colon or rectum, inflammatory bowel disease, and incomplete examination of the entire colon due to poor bowel preparation or technical difficulties. The study protocol was approved by the Institutional Research Ethics Board of Uijeongbu St. Mary's Hospital (IRB number UC14SISI0105). The study was conducted in accordance with the Helsinki Declaration.

### 2.2. Data Collection and Analysis

This retrospective analysis of prospectively collected data was performed using information from the endoscopy, clinical records, and pathology database system of Uijeongbu St. Mary's Hospital. Demographic data of patients included age, gender, smoking habits, comorbidities, personal and family history of CRC or other neoplasia, body mass index (BMI), and surgical history. Colonoscopy reports regarding characteristic of polyps (number and size of serrated polyps, polyp distribution, resection for polyps, and synchronous lesions) and diagnostic criteria met were collected.

Based on the WHO classification, serrated colorectal polyps were classified histologically as HPs, SSA/Ps with or without cytological dysplasia, or TSAs [7]. SSA/Ps are characterized by the presence of distorted and dilated crypts, especially at the base, along with excess serration near the base. The crypt often appears with a “boot” or “L” shape. TSAs are characterized by a protuberant or pedunculated growth pattern with distorted villiform configurations. They have columnar cells with abundant eosinophilic cytoplasm and centrally located elongated nuclei with an even chromatin pattern. The colon was divided into the distal (rectum, sigmoid colon, and descending colon) and proximal colon (splenic flexure, transverse colon, hepatic flexure, ascending colon, and cecum). Because information regarding family history is often subjective and not always reliable, none of the patients with SPS was diagnosed on the basis of the second WHO criterion (any number of serrated polyps proximal to the sigmoid colon in an individual who has a first-degree relative with serrated polyposis).

## 3. Results

**Figure 1:**
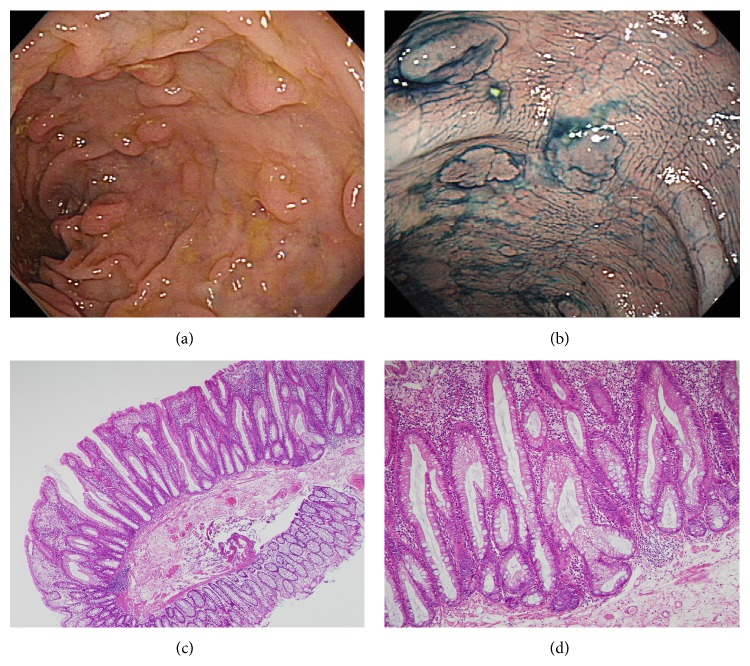
Representative example of serrated polyposis syndrome from patient number 1. Endoscopic findings showing multiple polyps in the sigmoid colon (a), endoscopic findings after indigo carmine spray (b), and histologic findings showing distorted and dilated crypts at the base along with excess serration near the base, original magnification ×10 (c) and ×100 (d).

**Figure 2:**
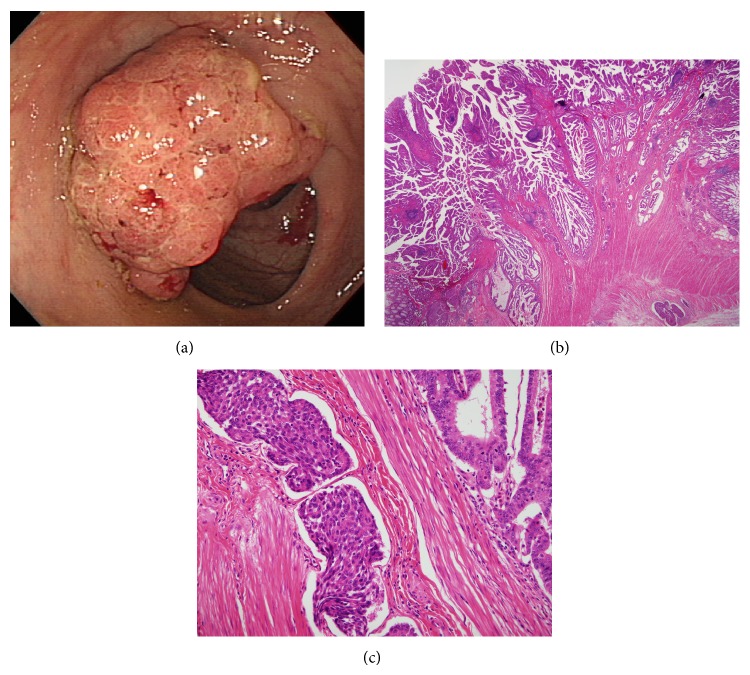
Representative example of serrated adenocarcinoma from patient number 11. Endoscopic findings showing 3.5 × 2.8 cm sized fungating mass in the descending colon (a), histologic findings showing crypts with dilation, serration, and irregular branching, original magnification ×12 (b), and tumor cells showing abundant cytoplasm, absence of necrosis, and eosinophilic cytoplasm, original magnification ×200 (c).

In total, 17,552 subjects underwent colonoscopies at Uijeongbu St. Mary's Hospital during the study period. Among them, 11 (0.06%) patients met the criteria for SPS (Table 1, Figure 1). Ten were males and one was female; their mean age at the time of diagnosis was 55.6 ± 9.6 (range 35–72) years. Demographic data are shown in Table 1. Of these 11 patients, 5 met WHO criterion 1, and 6 met criterion 3. Five patients (45%) had more than 30 serrated polyps, and the average size of the largest polyp was 22 mm. In total, 125 polyps were removed with conventional polypectomy or endoscopic mucosal resection in the 11 SPS patients. HPs comprised 36 (28.8%), SSA/Ps 49 (39.2%), TSAs 3 (2.4%), and tubular adenomas 37 (29.6%) of the 125 polyps. A pancolonic distribution of polyps was shown in nine (82%) patients, and proximal colon distribution was observed in the remaining two (18%) patients. Seven patients (64%) had synchronous advanced adenomas. There was no difference in the pathological characteristics of polyps of patients meeting criterion 1 versus those meeting criterion 3. One patient had coexisting SPS with CRC that was diagnosed at the initial colonoscopy. This CRC was serrated adenocarcinoma histopathologically (Figure 2). One underwent surgery and 10 underwent endoscopic resections.

In total, seven (64%) patients were current or former smokers. None had a family history of CRC or a first-degree relative with SPS. All of the patients underwent esophagogastroduodenoscopy (EGD). No patients had duodenal polyps or gastric adenomas on examination. Three patients (27%) had other extracolonic cancers or hematologic malignancies: one with hepatocellular carcinoma (HCC) associated with chronic hepatitis B, one with lung cancer, and one with myelodysplastic syndrome (MDS).

## 4. Discussion

SPS is a disease entity that is not well recognized, especially in Asian countries. Its genetic background and natural course have not been well characterized. In our study, we evaluated the clinicopathological characteristics of patients meeting the updated WHO criteria for the diagnosis of SPS. The prevalence of SPS in our study cohort was 0.06% (1 : 1,596). Previous reports from Western countries have shown variable results for the prevalence of SPS. Lockett and Atkin reported that the prevalence of SPS was relatively low (1 : 3,000; 0.033%) in the general UK population [12]. However, this result may have been underestimated because colonoscopy was performed only in patients with more than 20 polyps on flexible sigmoidoscopy. Orlowska et al. reported the frequency of SPS to be 0.056% (28/50,148) in asymptomatic patients who participated in colonoscopy-based CRC screening [13]. This result was similar to that of our study. However, recent studies reported that the prevalence of SPS was estimated between 1 : 151 and 1 : 294 in patients who underwent index screening colonoscopies based on fecal occult blood testing [8, 14]. These differences may be due to the differences in study design, patient populations, and other confounding factors. There have been few studies on the prevalence of SPS in Asia. Miwata et al. first reported the prevalence of SPS in Asia [15]. Of 73,608 Japanese patients who underwent colonoscopies in 14 hospitals, 10 (0.014%) met the WHO criteria for the diagnosis of SPS. However, the frequency of SPS in patients who underwent endoscopic resections was not so rare (0.38%) in this study. Vemulapalli and Rex reported that SPS was common in patients referred for resection of large sessile colorectal polyps, and it was frequently unrecognized [16]. Of the 529 patients, 20 were diagnosed as having SPS according to the WHO criteria, but only one patient was suspected by a referring physician. In our 11 patients with SPS, 5 were referred from local physicians for the resection of large colorectal polyps. All referred patients were undiagnosed at their first colonoscopy. A recent study demonstrated that patients with SPS underwent a median of four colonoscopies before satisfying the WHO criteria, and at least two pathologically confirmed serrated lesions are required to identify patients satisfying the WHO criteria for SPS [17]. Because SPS may be underdiagnosed due to lack of awareness, further studies are necessary to confirm its prevalence in Asians.

Previous studies showed that SPS has a near-equal gender distribution [9, 15, 18]. However, this study showed a strong male predominance in patients with SPS. The reason for this gender difference remains unclear. Recent studies from Western countries showed that most patients met WHO criterion 3 rather than criterion 1 [18, 19]. However, such a finding was not observed in our study or Japanese study, and this may be due to ethnic and/or racial differences [15]. Additionally, a recent study showed that there was no significant demographic, pathological, or molecular difference between patients meeting criterion 1 and patients meeting criterion 3, with the only exception of a greater likelihood of a family history of CRC and colonic polyps in patients meeting criterion 3 [19]. Further studies to compare clinical criteria with genotype are necessary.

The risk of personal and familial CRC is known to be increased in SPS. Previous studies showed that about 25–70% of SPS patients had CRC at the initial presentation or during follow-up [15]. A retrospective multicenter study showed that CRC was detected during surveillance in 5 of 77 SPS patients after a median follow-up of 1.3 years, and the cumulative risk of CRC under surveillance was 7% at 5 years [9]. In our study, only one patient (9%) had CRC at the initial presentation. This finding may be attributed to the short interval in colonoscopies performed and racial differences. The molecular basis of CRC development in patients with SPS remains unclear, but the importance of the serrated neoplastic pathway of carcinogenesis in SPS has been suggested [19, 20]. A recent prospective cohort study demonstrated that CRC was not detected in SPS patients during annual surveillance after endoscopic clearance of all polyps ≥ 3 mm, and the cumulative risks of detecting of CRC, advanced adenoma, or large serrated polyps (≥10 mm) were 0%, 9%, and 34%, respectively [21]. This result suggests that CRC development in SPS patients may be decreased by close endoscopic surveillance with removal of polyps ≥ 3 mm. However, further studies are needed to identify the most appropriate time interval for endoscopic surveillance and high risk patients requiring prophylactic surgery.

Additionally, emerging evidence shows that first-degree relatives of patients with SPS have an increased risk of developing CRC. Retrospective cohort studies reported an approximately fivefold increased incidence of CRC in first-degree relatives of patients with SPS compared with the general population [22–24]. Moreover, a recent study demonstrated that single screening colonoscopy in first-degree relatives of patients with SPS detected one or more significant polyps (43%), multiple polyps (9%), and SPS (14%) [25]. This suggests that a colonoscopy screening program for these individuals is necessary. None of the first-degree relatives of patients with SPS were diagnosed as having SPS in our study or a Japanese study [15]. However, the reason may be that colonoscopy was not performed in all first-degree relatives.

In the present study, 64% of patients with SPS were current or former smokers, which was higher than the prevalence of 46.2% of ever-smokers based on the data of the Korean National Health and Nutrition Examination Surveys [26]. A recent study showed that smoking at least 20 pack-years was strongly associated with SSAs of any and large sizes [27]. SPS patients also were significantly more likely to be current smokers than were either colonoscopy controls or population controls in another study [28]. Further studies should examine the effects of smoking in SPS patients in more detail.

Hereditary CRC syndromes (FAP and HNPCC) and hamartomatous polyposis syndromes (Peutz-Jeghers syndrome, juvenile polyposis syndrome, and Cowden syndrome) carry significant risks for extracolonic malignancies [29]. Although gastric neoplasia is a common disease in Korea, none of our patients had duodenal polyps or gastric adenomas on EGD. This result is similar to those from previous Western studies, suggesting that SPS patients may not be at increased risk of gastroduodenal polyps [30, 31]. Additionally, the present study showed that 27% had a history of extracolonic cancers or hematologic malignancies. Although HCC and lung cancer are common in the general population, SPS in MDS patients has not been reported. Further research is needed because studies regarding extracolonic tumors reported in SPS patients have shown inconsistent results [11, 31].

In conclusion, the prevalence of SPS in our cohort was comparable to that in Western populations. However, clinicopathological characteristics of patients with SPS showed some differences, including a strong male predominance and the WHO diagnostic criterion met. Additionally, SPS was almost unrecognized by referring physicians. Further large-scale multicenter studies are needed to confirm the prevalence of SPS in Asia. Considering the high risk of CRC, correct diagnosis and careful follow-up for SPS and colonoscopy screening for first-degree relatives of patients with SPS are necessary.

## Figures and Tables

**Table 1:** Demographic data and clinicopathologic characteristics of patients with serrated polyposis syndrome.

Patient number	Sex	Age	Colonoscopy indication	Number of polyps	Pathological characteristics of polyps	Size of the largest polyp (mm)	Treatment method	Diagnostic criterion^*^
1	M	59	Screening	>30	>20 HPs, 6 SSA/Ps, 8 TAs	25	ER	3
2	M	54	Screening	>30	>20 HPs, 1 SSA/P, 4 TAs	15	ER	3
3	M	50	Screening	>30	>20 HPs, 3 SSA/Ps, 2 TSAs, 4 TAs	20	ER	3
4	M	53	Positive FOBT	>20	>20 HPs, 2 SSA/Ps	15	ER	3
5	M	51	Screening	10	5 HPs, 4 SSA/Ps, 1 TSA	25	ER	1
6	M	58	Screening	17	5 HPs, 9 SSA/Ps, 3 TAs	15	ER	1
7	F	66	Anemia	>30	>20 HPs, 5 SSA/Ps, 3 TAs	20	ER	3
8	M	53	Screening	12	4 HPs, 5 SSA/Ps, 3 TAs	20	ER	1
9	M	35	Screening	6	2 HPs, 4 SSA/Ps	15	ER	1
10	M	72	Screening	20	6 HPs, 8 SSA/Ps, 6 TAs	30	ER	1
11	M	61	Screening	>30	>20 HPs, 4 SSA/Ps, 1 serrated adenocarcinoma	40	Surgery	3

^∗^Criterion 1: at least 5 serrated polyps proximal to the sigmoid colon with two or more of these being >10 mm; criterion 3: >20 serrated polyps of any size but distributed throughout the colon.

M: male; F: female; FOBT: fecal occult blood testing; HP: hyperplastic polyp; SSA/P: sessile serrated adenoma/polyp; TSA: traditional serrated adenoma; TA: tubular adenoma; ER: endoscopic resection.
